# Failure modes and effects analysis for clinical implementation of online adaptive radiotherapy: A systematic review

**DOI:** 10.1002/acm2.70749

**Published:** 2026-08-24

**Authors:** Deepikaa Balaji, Sonja Wegener, Sebastian Klüter, Sara L. Hackett

**Affiliations:** ^1^ Department of Radiotherapy The Netherlands Cancer Institute Amsterdam Netherlands; ^2^ Department of Radiotherapy Utrecht Medical Center Utrecht Netherlands; ^3^ Department of Radiotherapy and Radiation Oncology University Hospital Würzburg Würzburg Germany; ^4^ Department of Radiation Oncology Heidelberg University Hospital Heidelberg Germany; ^5^ Cancer Research @ UCC University College Cork Cork Ireland

**Keywords:** Failure Modes and Effects Analysis, Online Adaptive Radiotherapy, Quality Assurance

## Abstract

**Background:**

The accuracy of radiotherapy is limited by anatomical variations occurring over time scales ranging from sub‐seconds to days. Online Adaptive Radiotherapy (OART) addresses this by enabling daily plan adaptation based on real‐time imaging. While OART offers improved dose conformity, its dynamic, time‐constrained workflow introduces novel failure modes that challenge traditional quality assurance protocols.

**Purpose:**

This study aims to synthesize the existing literature on Failure Modes and Effects Analysis (FMEA) for OART to systematically catalog risks and identify mitigation strategies.

**Methods:**

A systematic literature search was conducted to identify studies applying FMEA to OART workflows. Eleven studies were included, covering MR‐guided (ViewRay MRIdian, Elekta Unity), CBCT‐guided (Varian Ethos), and MR‐enhanced C‐arm linac systems. To address heterogeneity in risk scoring methodologies (e.g., TG‐100 10‐point scales vs. 5‐point rankings), extracted failure modes were harmonized into a standardized three‐tier risk classification system (Class I: Low, Class II: Intermediate, Class III: High).

**Results:**

A total of 300 unique failure modes were identified, with 49.6 percent classified as high‐risk (Class III). Analysis revealed that the majority of high‐risk failures were concentrated in the online treatment delivery phase, specifically within human‐computer interactions and anatomical contouring steps.

**Conclusions:**

This study supports the development of tailored, robust QA frameworks that prioritize human factors and process consistency to guide safe implementation in diverse clinical settings.

## 1 INTRODUCTION

Radiotherapy treatment planning faces a longstanding challenge: the patient anatomy changes over the course of the treatment, especially in highly mobile regions such as the abdomen and lungs. Throughout a treatment course, which typically spans days or even weeks, anatomical or physiological changes can occur due to tumor shrinkage, organ deformation, respiration, bowel filling, or cardiac motion. These changes may happen both between fractions (interfractional variation) and/or within a fraction (intrafractional variation), and they can significantly affect the treatment accuracy. When the anatomy during treatment deviates from that used in the initial plan, the intended dose distribution may no longer align with the target volumes or adequately spare organs at risk (OARs).

This dynamic nature of anatomy has driven the development of Online Adaptive Radiotherapy (OART), a treatment strategy designed to account for such variations and optimize plan delivery accordingly.^1^ OART describes the process of modifying or re‐optimizing the treatment plan using imaging information acquired with the patient on the treatment couch, with the goal of mitigating the dosimetric impact of anatomical or physiological changes that occur over the course of therapy.^2^ This strategy can enhance target coverage and improve sparing of OARs compared to a non‐adaptive approach.

Technological innovations such as high‐quality imaging, deformable image registration, and motion management systems (e.g., respiratory gating) have particularly made OART clinically feasible and clinically relevant over the years.^3^, ^4^, ^5^, ^6^ Two main image‐guided technologies currently enable OART: Cone Beam Computed Tomography (CBCT) and Magnetic Resonance Imaging (MRI). CBCT is widely available and provides 3D anatomical information using X‐rays, while MRI offers superior soft‐tissue contrast without ionizing radiation, making it particularly suitable for tumors located in anatomically complex or mobile regions.^7^, ^8^

Within image‐guided adaptive radiotherapy workflows, two principal adaptation strategies are commonly used. The first is a shift‐based method, which involves applying a rigid registration between the planning and daily images to align the original anatomy with the patient's current position. This registration is typically followed by a (virtual) couch shift, enabling the original treatment plan to be delivered without modification. This approach assumes that the anatomical changes are subtle enough that the contours and associated treatment plan are still valid. The second strategy involves re‐contouring the target and OARs on the daily image and generating a completely new treatment plan to accommodate anatomical deformations or shifts. Although the latter is more time‐consuming, it provides greater dosimetric accuracy by accounting for tumor shrinkage, organ deformation, or displacement of critical structures.^9^

While OART directly addresses the limitations of conventional radiotherapy, it also presents specific clinical and operational challenges. OART does not follow a universal protocol. Practices vary between institutions, depending on available equipment, site‐specific workflows, and individualized patient requirements, such as anatomical complexity or proximity of critical structures. These variations introduce new risk points unique to each implementation.^10^ The compressed timeline from imaging to treatment requires streamlined workflows, efficient software integration, and rapid clinical decision‐making. As Noel et al.^11^ emphasize, these characteristics introduce unique risks that must be managed with dedicated safety protocols, since traditional Quality Assurance (QA) strategies like phantom‐based pre‐treatment QA are often incompatible with the time‐sensitive nature of online adaptation.

To address these risks, Failure Modes and Effects Analysis (FMEA) has emerged as a systematic, proactive tool to identify and prioritize failure points in complex clinical processes. FMEA assigns the occurrence, severity and detectability to each failure mode, helping staff recognize high‐risk steps and target them for mitigation. In the context of OART, FMEA plays a critical role in mitigating risks associated with adaptive workflows.^12^

However, while several institutions have conducted FMEA studies tailored to their specific OART workflows, there is no consolidated evaluation synthesizing their findings. As a result, valuable insights into shared failure modes and mitigation strategies remain fragmented. Inspired by the work of O'Daniel et al.,^13^ who consolidated and ranked failure modes to identify those most critical to treatment safety, this study aims to systematically evaluate published FMEA studies on OART, extract and aggregate failure modes, rank them by relative risk, and highlight recurring challenges and potential mitigation strategies.

In summary, the goal of this study is to provide a clear, evidence‐based overview of the most critical risks associated with OART, offering practical guidance to clinical staff, safety managers, and technology developers. Ultimately, this work aims to support the development of more consistent, streamlined, and proactive QA strategies to strengthen patient safety and workflow reliability in the evolving field of OART.^14^

## 2 METHODS

### 2.1 Literature search and study setting

A structured literature search was performed in the PubMed database using the keyword combination ((FMEA) OR (Failure Modes and Effects Analysis)) AND (Online Adaptive Radiotherapy). Titles and abstracts were screened to include seven studies that (1) focused specifically on online adaptive radiotherapy (OART) and (2) reported a defined list of failure modes, with or without corresponding occurrence and severity scores. Through reference screening, four additional articles that met these criteria were included.

In total, eleven relevant articles were included (Table I). Nine of these presented failure modes with corresponding occurrence (O) and severity (S) scores. The remaining two articles listed failure modes without scoring; for these, scores were assigned by one author using a 5‐point scale (refer to Table I of Kluter et al.^15^ 2021 for the applied criteria) and then reviewed by a second author. Additionally, incident reports related to adaptive workflows were reviewed from the SAFRON (Safety in Radiation Oncology) and RO‐ILS (Radiation Oncology Incident Learning System) databases^16^.

Two additional articles, while not meeting the criteria, contributed valuable contextual insights. Noel et al. introduced a process‐oriented framework for FMEA in radiotherapy, providing the conceptual basis for mapping adaptive workflows and risk propagation in this study. Green et al.^17^ was used to characterize the clinical workflow for OART, providing the reference framework against which failure modes were categorised.^11^

The selected studies represent eight institutions across Germany, Switzerland, the United States, and Japan, each applying FMEA principles within their clinical environment. Notably, two institutions conducted paired investigations, offering longitudinal perspectives on the evolution of OART workflows.

At the University Hospital Würzburg (Germany), Wegener et al. published a prospective FMEA of an AI‐driven CBCT‐based adaptive workflow on the Varian Ethos system, followed by a one‐year re‐evaluation of the same process.^18^, ^19^ The second study revisited the original risk framework using clinical experience, thereby validating and refining the earlier analysis.

Similarly, at the University Hospital Zurich (Switzerland), Schüler et al. (2021) reported their institution's initial experience implementing the ViewRay MRIdian MR‐Linac using an FMEA framework.^20^ Wilke et al. (2025) later expanded this framework to a novel MR‐enhanced daily adaptive workflow on a Varian TrueBeam C‐arm linac combined with a diagnostic MR simulator.^21^ While not a direct clinical continuation, the latter demonstrated the progression of MR‐guided safety principles to a more accessible system configuration.^22^

A third paired investigation was identified examining the Elekta Unity MR‐Linac. Liang et al. conducted independent FMEAs for two distinct online adaptive workflows: adapt‐to‐position (ATP) and adapt‐to‐shape (ATS). As these workflows differ fundamentally in structure and decision points, they provide complementary coverage of failure modes across the major adaptive strategies implemented on the same platform.^23^, ^24^

Together, these institutional pairs reflect complementary trajectories in the literature: longitudinal validation of risk analyses (Würzburg), methodological translation across hardware platforms (Zurich), and workflow‐specific risk characterization within a single platform (Unity MR‐Linac). The evaluated studies encompassed diverse OART workflows, including the ViewRay MRIdian (*n* = 3), Varian TrueBeam (*n* = 1), Varian Ethos (*n* = 5), and Elekta Unity (*n* = 2), representing the major systems currently applied in clinical adaptive radiotherapy.

### 2.2 Clinical workflow of online adaptive radiotherapy

**FIGURE 1:**
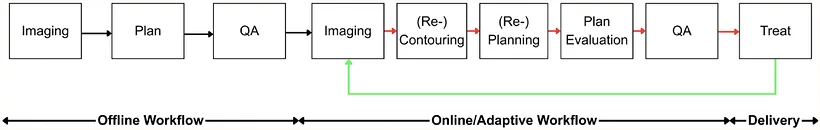
Online adaptive process with major workflow steps. The green arrow represents the repetition of the workflow for each online adaptive treatment fraction adopted from Green et al.

The implementation of OART involves a structured workflow combining advanced imaging, rapid treatment planning, and integrated QA measures. The study by Green et al. characterizes the clinical workflow for OART by distinct sequential processes: *Simulation, Treatment Planning, Localization, Imaging, Assessment, Replanning, Quality Assurance (QA), and Delivery* (Figure 1).

Volumetric Imaging (with MRI or CBCT), is performed to visualize patient anatomy. During the assessment phase, the user determines whether adaptation is required. Predetermined tolerances on anatomical variations can guide the decision to initiate adaptation.

New contours are then generated on the daily images, either via propagation from the reference dataset to the new one or autosegmentation. However, these contours often require evaluation and manual editing. Manual contour editing is not only time‐intensive, it is also vulnerable to inter‐observer variability and user error, necessitating careful review by the clinical team. Replanning follows contour approval and involves generating a new treatment plan based on the updated anatomy. This may include plan re‐optimization or adjustments to the dose distribution, typically performed in a time‐constrained environment using dedicated planning systems. Errors during this step can affect both target coverage and normal tissue sparing, especially when performed under pressure.

QA is a necessary and time‐sensitive step in the OART workflow, performed immediately prior to treatment delivery. Since the patient remains on the treatment couch throughout the adaptive process, traditional QA methods, such as phantom‐based dose verification are not feasible. Instead, clinics rely on in‐silico tools and automated plan integrity checks to verify the adapted plan before beam‐on. These QA steps are typically integrated into the treatment planning system and must be completed quickly to prevent additional delays or anatomical changes.

Finally, adaptive treatment delivery is performed, often incorporating intra‐fraction motion management techniques such as breath‐hold and/or beam gating to maintain anatomical stability throughout dose delivery. In each adaptive fraction, the clinical team may document key aspects of the session such as the rationale for adaptation, contour modifications, and plan approval to ensure traceability and treatment safety.

For the purpose of this evaluation, this generalized workflow was adopted as a reference framework for cross‐study comparison. While the exact implementation of OART can vary between institutions and treatment systems, the essential sequence of adaptive steps (i.e., imaging, assessment, contouring, replanning, QA, and delivery) remains consistent. Establishing this common structure enables a unified interpretation of the published FMEAs and supports the identification of shared risk patterns across different adaptive platforms.

### 2.3 Study methodology

Selected articles were read iteratively by a team of researchers and medical physicists with active clinical and research involvement in OART workflows across both CBCT‐based and MR‐Linac platforms. Two authors performed the primary data extraction, variable identification and analysis and agreement was defined as consensus between both following iterative discussion of any differing interpretations. The list of variables finalized for analysis were “Main Process”, “Sub‐Process”, “Failure Mode”, “Cause of Failure”, “Occurrence”, “Severity”, “System (Viewray/Unity/Ethos)”, “Mitigation Strategy”. All selected articles were analyzed using a predetermined datasheet with above variables. No SAFRON reports were found to be specifically relevant to OART. However, two ASTRO RO‐ILS reports were found to be relevant to OART workflows. In both cases, relevant insights were already captured and analyzed in the included FMEA studies and were therefore not extracted separately in this study.

### 2.4 Ranking methodology

For this study, only risks specifically related to the online adaptive radiotherapy (OART) process were considered.^13^ Five studies used the TG‐100–based 1–10 scale for severity and occurrence scores,^25^ while others employed simplified 1–5 risk matrices as shown in Table 1. Since the included institutions employed heterogeneous approaches to risk scoring, a harmonization strategy was required. To enable meaningful cross‐study comparison, ranking criteria were adapted from Klüter et al. 2021^15^ and standardized across the diverse scoring systems used by different institutions following the approach of Kornek et al. 2024^26^ All outcomes were then grouped into three qualitative categories: Class I (low), Class II (intermediate), and Class III (high). It is important to note that “Class III” in this context does not represent an identical numeric threshold across institutions but rather a relative classification of high‐risk events within each institution's scoring framework. This approach ensured comparability while acknowledging differences in the underlying scoring philosophies across studies.

**TABLE 1:** A summary of included FMEA studies categorized by device, system, source, and scoring method.

Device	System	Source	FMEA scoring method used
MR‐linac	MRIdian (ViewRay)	[i] Klüter 2021	5‐Point Ranking Scale
	MRIdian (ViewRay)	[ii] Nishioka 2022	TG‐100 10‐Point Scale
	MRIdian (ViewRay)	[iii] Schüler 2022	Non‐TG‐100 10‐Point Scale
	Unity (Elekta)	[iv] Liang 2023	5‐Point Ranking Scale^*^
	Unity (Elekta)	[v] Liang 2025	5‐Point Ranking Scale
MR‐Enhanced Linac	TrueBeam (Varian)	[vi] Wilke 2025	TG‐100 10‐Point Scale
CBCT	Ethos (Varian)	[vii] Rahman 2024	TG‐100 10‐Point Scale
	Ethos (Varian)	[viii] Wegener 2024a	5‐Point Ranking Scale
	Ethos (Varian)	[ix] Wegener 2024b	Non‐TG‐100 10‐Point Scale
	Ethos (Varian)	[x] Colbert 2025	TG‐100 10‐Point Scale
	Ethos (Varian)	[xi] Zheng 2025	5‐Point Ranking Scale

* Occurrence and/or Severity scores were estimated by an author familiar with the Elekta Unity system based on the failure mode descriptions.

Each study's failure modes were ranked independently, as the FMEA varied in how severity and occurrence were assessed. In all cases, risk classes were derived from two‐dimensional matrices mapping severity against occurrence, in alignment with institutional risk tolerance strategies.^15^ To ensure transparency and allow readers to interpret each study's framework appropriately, the original risk matrices or scoring schemes used in each paper are included in the Appendix (Tables A1, A2 and A3).

## 3 RESULTS

A systematic Evaluation of the selected literature and incident reports yielded a total of 300 unique failure modes associated with OART workflows. (Refer to ’FMEASystematicEvaluatoin.xlsx’ from Appendix B for the compiled list of failure modes) These were drawn from studies covering various systems, including ViewRay, Varian Truebeam and Elekta Unity Linacs and the Varian Ethos CBCT‐based platform. A key finding from the compiled data is that the process of OART is regarded as having a high inherent risk. Notably, 49.6 percent of these failure modes were assigned a high‐risk classification (Risk Class III or equivalent) by the original authors, indicating strong consensus that online adaptation introduces substantial safety risks across institutions and technologies (Table 2).

**TABLE 2:** Risk matrix of failure modes by occurrence (O) and severity (S) ranking, with class‐wise risk distribution.

O \ S	1	2	3	4	5
**1**	10	11	14	5	2
**2**	16	27	28	22	10
**3**	1	14	17	15	12
**4**	1	7	19	50	8
**5**	1	6	2	2	0

Risk class	Number of failure modes
Class I (low risk)	40
Class II (moderate risk)	111
Class III (high risk)	149
**Total**	**300**

To better understand the nature and distribution of these risks, the compiled failure modes were analyzed in three ways: (1) by identifying recurring failure mode themes across studies, (2) by mapping failure modes to specific workflow stages, and (3) by evaluating technology‐specific risks across different imaging systems.

### 3.1 Recurring failure modes and patterns

To facilitate a clear analysis, failure modes that were frequently repeated across different studies and institutions were identified and consolidated in order to identify overaching patterns in the raw data. Analysis of the compiled failure modes reveals several recurring themes. These patterns are not specific to any single technology but rather appear to be inherent to the complexity of the adaptive process itself. Four primary themes were identified. Across 300 extracted failure‐mode entries, human/process factors (*n* = 144) and anatomical delineation (*n* = 124) dominated, followed by data‐quality issues (*n* = 35) and anatomical variability (*n* = 15).

*Human‐Computer Interaction and Process Adherence*: Human factors represent the most prominent category of high‐risk failure modes. These include errors of selection, such as loading an incorrect patient plan or choosing a wrong reference scan; errors of oversight, like failing to notice incorrect automated contours or dose constraint violations; and errors of judgment, particularly when making complex clinical decisions under significant time pressure.

*The Centrality of Anatomical Delineation*: Contouring was consistently identified as a key area of vulnerability, both in the preparatory and online phases. The accurate delineation of the target volume (GTV/CTV) and OARs is a foundational step, and errors in this task are linked to a number of high‐risk failure modes.

*Quality of Data*: A notable subset of failure modes is associated with data fidelity, which highlights that the quality of the output (adapted plan) is fundamentally dependent on the integrity of the input data. This creates a ’domino effect’ where a single upstream error cascades through the workflow. For example, an initial error like generating countours that extend beyond the body can corrupts the plan's spatial foundation, invalidating all subsequent steps.

*Anatomical Variability*: The inherent biological and anatomical changes that OART is designed to manage also represent an operational challenge. Pronounced interfractional variation (such as patient movement due to bladder filling or gas pockets) can increase the complexity and duration of the daily adaptation, potentially heightening the likelihood of other failures related to contouring accuracy, clinical decision‐making and patient tolerance of the procedure.

### 3.2 Process‐based analysis

**FIGURE 2:**
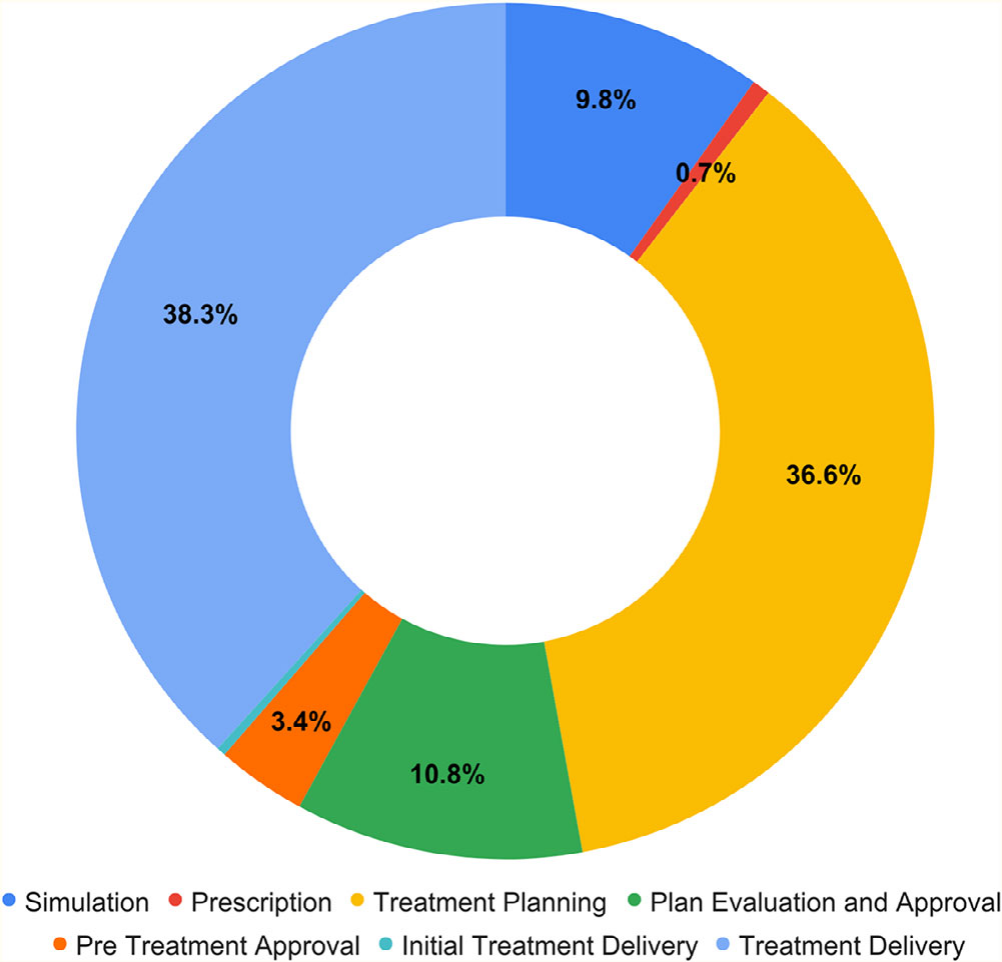
Distribution of the 300 compiled failure modes across the defined OART activities, expressed as a percentage of the total.

To understand where these vulnerabilities are most concentrated, the failure modes were categorized according to the activity in which they occurred within the overall treatment workflow. The distribution was non‐uniform, with a clear concentration of high‐risk scenarios in the following phases as depicted in Figure 2.

*Online Treatment Delivery*: As expected, this was considered to be the most risk‐prone phase as it involves all real‐time, on‐table clinical activities. This phase represents the most complex and time‐sensitive part of the workflow, making it the primary locus of risk. Key sub‐processes within this stage, including Online Adaptation, Imaging and Online Re‐Contouring are associated with the highest frequency of high‐scoring failure modes. The events in this phase are notable because their consequences are immediate, occuring at the final stages before dose is delivered.

*(Reference) Treatment Planning*: Conducted offline, this step is identified as the next crucial area. As the stage where the core planning objectives such as dose prescription, normalization and dose constraints are established, it is a critical control point. Vulnerabilities here, such as the use of incorrect normalization values or planning templates, can introduce systemic errors that may propagate throughout the entire treatment course.

*Plan Evaluation & Approval*: This stage serve as a key decision‐making step in the workflow, where staff evaluate the adapted plan and confirm acceptability for treatment delivery. While associated with fewer failure modes, these processes are designed to catch critical errors. Failures at this stage, such as incorrect patient or plan identification, represent a breakdown of fundamental safety protocols and are therefore associated with high‐severity outcomes.

A selection of high‐risk (Risk Class III) failure modes specific to online adaptive radiotherapy workflows, categorized by process step has been presented in Table 3. Failure modes were selected based on a severity score of ≥ 4 (corresponding to S ≥ 7 on the TG‐100 scale, indicating potentially serious dosimetric or clinical consequences), representing the most clinically impactful risks at each workflow stage. Table 1 prioritizes failure modes that appeared across multiple studies, as their recurrence across institutions indicates consistent clinical relevance. Scores are reported as (O/S) based on 5‐point ranking scale; refer to Table 1 and Table A1 of Appendix A for scale definitions. For literature sources that reported both failure modes and their associated consequences, both elements were extracted and included directly in the table. In cases where the source reported failure modes only, the corresponding potential consequences were inferred based on the clinical experience and expertise of the review authors.

**TABLE 3:** A selection of high‐risk (Risk Class III) failure modes with a severity score of ≥ 4 (corresponding to S ≥ 7 on the TG‐100 scale) in OART workflows, categorized by process step.

Process Step	Failure Mode	Description of effect(s)	Scores (O/S) [Source]^*^
**Pre‐Treatment Approval**	Unsuitable patient scheduled for adaptation; Adaptation not possible	Patient unable to complete adaptive session; session may need to be terminated or a new reference plan may be needed	III(2/5) [viii]
	MRI non‐compliance or breathing problems due to poor patient selection.	Suboptimal image quality; adaptation may proceed on unreliable anatomical information.	III(4/4) [i]
**Treatment Planning**	Wrong normalization (e.g., inconsistency between total dose and fractions).	Systematic dose error propagated across all fractions. Normalization error results in unsuitable plan (e.g., very wrong dose distribution)	III(4/3) [viii], III(4/5) [ii]
	Wrong dose constraints due to use of the wrong template.	Suboptimal plan quality	III(4/4) [vii, viii], III(4/5) [ii]
	Inappropriate optimization parameter or wrong structure used for optimization.	Suboptimal dose distribution; potential target underdosage or OAR overdosage.	III(4/4) [ii]
***Online Workflow***			
**Imaging**	Very wrong localization due to misunderstanding of target location.	Incorrect anatomy used as basis for adaptation; geometric miss of target possible.	III(1/5) [ii]
	Major interfractional changes leading to quality loss of sCT	Incorrect dose calculation	III(4/4) [viii]
**(Re‐)Contouring**	Incorrect/inadequate delineation of critical structures due to time pressure	Inaccurate dose distribution. On systems requiring bulk density overrides, incorrect delineation additionally compromises the electron density map, propagating errors into dose calculation	III (4/4) [ii]
	Patient/anatomical movement during online preparation.	Contours no longer reflect current anatomy at time of delivery even with additional imaging leading to dose deviation.	III (4/4) [i, ii]
	Lengthy contouring or dose calculation due to unavailable staff member	Time delays lead to increased intrafractional motion and therefore reduced plan quality	III(3/5) [viii]
	Wrong adaptation of GTV due to poor imaging or new physician.	Incorrect target volume; risk of geographical miss or unnecessary OAR irradiation.	III(4/4) [ii]
	Incorrect contours due to adaptation on old, static contours.	Inadequate plan quality	III(4/4) [viii]
	Error in automatically generating structure, resulting in adaptation on erroneously derived structures	Incorrect dose distribution	III(4/3) [viii]
**(Re‐)Planning**	Adaptation not based on best possible contours due to poor handover.	Patient details not known to Advanced Adaptor leading to suboptimal plan quality; clinical intent not fully captured in adapted plan.	III(4/5) [viii]
	No adaptation executed	Hot spots oversight or dose constraint violations carelessly permitted.	III(4/4) [i]
	Treatment course prolonged due to patient movement/bladder too empty to start, making adaptation difficult.	Adaptation delay resulting in delayed or missed treatment fraction.	III(3/5) [viii]
	Long contouring process due to insufficient training or experience	Delayed treatment, inadequate margins and therefore incorrect dose distribution	III(4/4) [viii]
**Plan Eval. & Approval**	Error in plan description in planning/treatment directive	Sub‐optimal delivery or incorrect dose	III(4/4) [ii]
	Loading the wrong plan when resuming after treatment interruption.	Non‐adapted or incorrect plan delivered; dosimetric error for that fraction.	III(3/5) [ii]
	Instead of the optimized plan, the old one is executed.	Patient treated with reference plan despite anatomy having changed, leading to suboptimal plan quality for that fraction.	III(4/4) [i]
	Poor plan quality due to time constraints.	Suboptimal plan accepted under pressure; compromised clinical outcome.	III(4/4) [i]
	Incorrect dose prescription due to incorrect scaling of multiphase plan.	Incorrect plan optimized and delivered.	III(2/5) [iv]
	A non‐approved plan is prepared for treatment.	Suboptimal plan delivered.	III(4/4) [i]
	Incorrect density assignment	Dose calculation error.	III(4/4) [i], III(4/3) [vii], II(3/2) [iii]
**Treatment Delivery**	Server/network problems lead to device unavailability.	Adaptive treatment cannot proceed likely due to device switch and no regular maintainence of equipment; fraction delayed or missed.	III(2/4) [viii]
	Dose constraint violation carelessly permitted.	OAR overdosage accepted and delivered without appropriate clinical justification.	III(4/4) [i]
	Plan variant confusion.	Wrong plan variant selected for delivery; incorrect dose to patient.	III(3/4) [ix]

* Note: Roman numeral references in the [Source] column correspond to the study citations listed in Table 1.

### 3.3 System‐based analysis

While many failure modes are common to all OART workflows, the underlying imaging technology introduces distinct, system‐specific vulnerabilities. A particularly instructive difference between platforms is the method used to generate the density grid for dose calculation either through deformable CT‐to‐MR registration combined with manual bulk density overrides, through electron density assignment from a reference CT, or directly from CBCT‐derived synthetic CT. Analysis of the compiled failure modes reveals that each method gives rise to a qualitatively different pattern of risk.

*MR‐Guided Systems (ViewRay and Elekta Unity)*: The failure modes unique to these systems are predominantly related to the magnetic field and the imaging modality itself. High‐risk failures identified in the literature include patient‐related issues such as MRI non‐compliance or breathing problems due to poor patient selection, which are critical due to the longer acquisition times and enclosed gantry.^15^ Technology‐specific imaging failures are also prominent, such as poor GTV visibility or tracking on the MR cine images and suboptimal positioning of patient or coils leading to poor MR quality.^20^ These highlight a dependency on image quality that directly impacts both visualization and motion monitoring tracking capabilities.

Our compiled failure modes also reveal that density‐related risks were consistent among the ViewRay studies, a risk attributable not to the system itself but to the CT‐to‐MR registration step. On ViewRay systems, the density grid is derived through deformable CT‐to‐MR registration combined with manual bulk density overrides applied to air cavities and regions of registration mismatch.^27^ The cognitively demanding nature of this process means that these risks are predominantly human‐driven such as including forgotten visual density checks, incorrect override application, and erroneous plan parameters and are primarily mitigated through checklists and independent second checks. On the Elekta Unity, electron density is instead assigned from the reference planning CT, introducing a different failure pattern: an incorrect ED assignment at the planning stage propagates silently across all subsequent fractions, representing a low‐visibility upstream risk.

*MR‐Enhanced Systems (Varian TrueBeam with Siemens MR Simulator)*: In MR‐enhanced adaptive workflows such as the approach described by Wilke et al.^21^ (2025), an MRI is acquired on a standalone MR simulator prior to treatment, and patients are transferred to a conventional linac for delivery. The primary risks in this setup arise from the decoupled nature of imaging and treatment specifically, registration and transfer errors, workflow delays, and potential misalignment during patient repositioning. The generation and validation of synthetic CTs (sCTs) from MR data are also critical, as inaccuracies can propagate into the adaptive planning and dose calculation stages. While this workflow provides the soft‐tissue contrast benefits of MRI at lower cost than MR‐Linacs, it introduces additional logistical and registration‐related failure modes.

Similar to ViewRay, the density grid on this platform relies on deformable CT‐to‐MR registration, and the associated failure modes follow the same human oversight pattern such as incorrect registrations and wrong density parameters underlining that this vulnerability is inherent to the registration‐based density approach rather than specific to any single vendor.

*CBCT‐based Systems (Varian Ethos)*: The primary technology‐specific risks center on the quality of the daily imaging and its use in dose calculation. In earlier (pre‐HyperSight) Ethos versions, dose was not computed directly on a CBCT‐derived electron density map, since the daily CBCT did not provide sufficient HU fidelity; instead, the planning CT was deformably registered to the daily CBCT and its Hounsfield units propagated into the daily anatomy to generate a synthetic CT (sCT) on which dose was calculated.^28^ The literature identifies critical failure modes in this process, such as poor‐quality sCTs arising from major anatomical changes or gas pockets, where errors in the underlying deformable registration propagate directly into the dose calculation.^19^ It is important to note that most of these reported failure modes originate from clinical experience where CBCT image quality and HU fidelity were more limited. The workflow is also vulnerable to artifacts from positioning aids (e.g., perforated boards) that can degrade CBCT image quality and, consequently, the accuracy of the adaptation.^18^ Critically, Colbert et al. (2025) identified that on systems without the HyperSight upgrade, the deformable registration of the planning CT to the daily CBCT which underpins dose computation cannot be inspected by the user, meaning errors may propagate silently into the adaptive plan.^29^ Unlike MR‐based systems where density failures are primarily human‐driven, Ethos density risks are fundamentally data quality‐driven, triggered by anatomical factors independently of operator actions.

## 4 DISCUSSION

Our first analysis revealed that the most consistent vulnerabilities lie at the intersection of human–computer interaction, particularly under time constraints. This is largely due to the cognitive load placed on the clinical team during time‐sensitive tasks. These findings align with broader concerns raised in the human factors literature. For instance, Wong et al.^30^ (2024) demonstrated how cognitive overload during on‐table planning can lead to decision‐making flaws such as anchoring and inattentional blindness. Supporting these observations, Wilke et al. (2025) identified imprecise work and loss of attention due to environmental distractions, such as crowding and noise in the control room as a recurrent human‐factor risk during online adaptive sessions.^21^ Although seemingly minor, such conditions exacerbate cognitive fatigue and can compromise decision accuracy at critical workflow stages. Collectively, these insights underscore the importance of incorporating cognitive ergonomics, structured task allocation, and targeted training into future OART protocol design. In line with these perspectives, a recent study by Rajpurkar et al.^31^ (2025) further emphasize that optimal human–AI collaboration achieved through clear task separation rather than integration may help mitigate automation bias and enhance workflow safety.

At the same time, it is important to recognize that innovation does not solely introduce new risks but can also reduce existing ones. Colbert et al.^29^ (2025) demonstrated that transitioning from conventional IGRT to adaptive workflows decreased the overall risk profile, with median RPN scores reduced through iterative plan review and automation‐assisted checks. This reinforces that new technologies such as AI‐driven automation and adaptive QA may simultaneously introduce and resolve different categories of failure modes underscoring the need for balanced, evidence‐based evaluation frameworks when integrating novel tools into clinical workflows.

The process‐based analysis provided a clear map of the OART workflow, highlighting that while treatment planning is crucial, the online environment of the daily treatment session is the most critical area for risk awareness and management. This underscores the need for targeted mitigation strategies in online adaptation, where the immediacy of decisions compounds the risk of error. Planning and approval phases, while associated with fewer immediate failures, contain upstream errors that can propagate downstream if undetected.

The density‐related failure modes identified in the system based analysis of this study highlight a broader opportunity for workflow innovation. For MR‐guided systems, the elimination of the planning CT through MR‐only planning workflows would remove the CT‐to‐MR registration step entirely, and with it, the associated human‐driven failure modes. While clinical implementation remains challenging, recent developments in sCT generation from MRI suggest this may be a promising direction.^32^ Similarly, for CBCT‐based systems, advances in CBCT reconstruction aimed at achieving CT‐quality images directly from daily CBCT can bypass sCT generation and its associated data quality vulnerabilities. On platforms such as HyperSight (Varian Medical Systems), advanced iterative reconstruction now achieves HU accuracy comparable to fan‐beam CT and is already used clinically for direct dose calculation in adaptive workflows,^33^ while deep learning–based reconstruction offers a further avenue towards this goal.^34^

The findings of this study are consistent with those of a recent independent multi‐centre FMEA by Osman et al.^35^ (2026), who prospectively identified high‐risk oART failure modes across five UK centers using a modified Delphi approach. Both studies converge on contouring, dose calculation, and plan evaluation as the highest‐risk workflow steps, lending cross‐methodological validity to these findings. While Osman et al. provide a prospectively developed consensus framework specifically designed to inform clinical trial QA, the present study complements this by synthesizing a broader body of published institutional experience encompassing 300 failure modes across 11 studies and by including MR‐enhanced C‐arm linac workflows, which Osman et al. 2026 identify as a gap requiring future investigation.

Furthermore, we opted for a qualitative risk matrix rather than Risk Priority Numbers to enable comparison across studies with different scoring methodologies. A qualitative ranking system helps mitigate this variability and offers clearer prioritization. As Klüter et al. highlighted, risks with identical RPNs can differ significantly in clinical relevance. That is, a risk with critical severity and very rare occurrence is not equal to a risk with small severity and occasional occurrence. The matrix‐based approach also proved more intuitive and accessible for interdisciplinary teams unfamiliar with traditional FMEA scoring.

However, several considerations should be kept in mind when interpreting these results. Source heterogeneity posed a challenge: the included studies varied in their scoring methodologies and approaches to identifying failure modes. A “Class III” event in one study does not necessarily correspond to a “high‐risk” event elsewhere. While our qualitative standardization enabled meaningful cross‐study comparison, it inevitably abstracted away some contextual nuances that shape how risk is perceived. For example, Zheng et al. (2025) adopted a purely observational, practice‐based approach, reporting only failure modes that actually occurred, whereas other studies extrapolated additional hypothetical risks within prospective FMEAs.^36^ This distinction between observed and anticipated risks illustrates the diversity of analytical philosophies within the literature. In addition, factors such as institutional experience, program maturity, and team familiarity with adaptive workflows strongly influence how failure modes are scored. Centers with limited OART experience often rated a large fraction of failure modes as high‐risk, whereas mature programs supported by procedural confidence and accumulated incident data tended to classify the same events as moderate or low. Thus, FMEA scoring should not be viewed as an absolute measure of hazard severity but rather as a context‐dependent reflection of institutional expertise, analytical approach, and local safety culture.

Second, this study deliberately focused on OART‐specific risks and excluded general radiotherapy hazards. Several foundational works included multi‐institutional and process‐level analyses, as well as broader framework guidance from AAPM TG‐100 provide important context for managing such baseline risks within broader radiotherapy workflows.^25^, ^37^, ^38^, ^39^ Thirdly, there is an uneven distribution of included studies across platforms, with five studies of the Ethos platform, three of the ViewRay platform, two of the Unity platform and one of the TrueBeam with MR‐enhanced workflow. This uneven distribution means that certain risks may be over‐represented in the analysis, and precludes a quantative comparison of risk profiles between systems. Nonetheless, qualitative differences in failure mode patterns are evident. Finally, the dynamic nature of the field means that this evaluation is a snapshot in time. New software updates, hardware improvements, and the growing use of artificial intelligence for tasks like auto‐segmentation will undoubtedly alter the risk landscape, potentially mitigating some of the failures identified here while introducing new ones.

Based on the concentration of risks identified, future work should focus on developing and validating targeted mitigation strategies, particularly for the online treatment delivery phase. There is a clear need for standardized yet flexible QA checklists and decision‐support tools that can help clinical teams navigate the high‐pressure online environment. Research into human factors engineering could further optimize the user interfaces of OART systems to reduce cognitive load and minimize selection or oversight errors.^31^ Furthermore, the development of automated, AI‐driven QA tools for tasks such as contour verification and plan integrity checks could provide a crucial safety layer.^29^ We recommend that institutions implementing OART not only perform a baseline FMEA but also engage in a continuous, iterative process of re‐evaluation, as suggested by Wegener et al., to refine their protocols as they gain clinical experience.

## 5 CONCLUSION

This systematic evaluation attempted to provide a comprehensive map of the risk landscape associated with an advanced treatment modality like OART. The analysis confirms that the OART workflow, particularly the online treatment delivery phase, is a domain of high‐stakes activities where the potential for critical failure is concentrated. The most significant vulnerabilities identified are not related to isolated equipment malfunctions but are deeply embedded in the complex interplay of human factors, process adherence, and data integrity. While the FMEA framework provides a systematic method for risk identification, this evaluation revealed clear institutional variability in how these risks are scored and prioritized. This underscores a critical insight: the perception and management of risk in OART are highly context‐dependent. Factors such as the specific imaging technology employed (MR‐Linac vs. CBCT), institutional experience, and local safety culture can fundamentally shape the risk profile.

Therefore, the ultimate conclusion of this study is that a “one‐size‐fits‐all” approach to OART safety is neither feasible nor optimal. The development of a single, universal checklist or protocol would fail to address the unique vulnerabilities inherent to each clinical environment. Instead, the true value of this collected analysis is to serve as a foundational resource. It provides a comprehensive catalog of potential failure points that clinical teams can use as a starting point to guide their own local, context‐specific risk assessments. By understanding the common vulnerabilities, institutions can develop tailored and robust QA programs that effectively mitigate the risks relevant to their own staff, patients, and technology, thereby ensuring the safe and successful delivery of this transformative treatment modality. Ultimately, this work contributes to the creation of safer, more resilient OART ecosystems by identifying the highest‐risk activities and contextualizing them within a structured safety framework.

## AUTHOR CONTRIBUTIONS

Sara L. Hackett conceived of the presented idea. Deepikaa Balaji developed the idea and carried out the systematic review and analysis in discussion with Sebastian Klüter, Sonja Wegener and Sara L. Hackett. Deepikaa Balaji wrote the manuscript, and the other authors reviewed and edited the manuscript, together with Deepikaa Balaji.

## CONFLICT OF INTEREST STATEMENT

SK has received speaker fees and travel reimbursement from Accuray and Siemens Healthineers. The rest of the authors declare no conflicts of interest.

## Supporting information



Supporting Information

## APPENDIX A

### Risk classification framework

**TABLE A1:** Risk matrix of failure modes by occurrence (O) and severity (S) ranking, with classwise risk distribution for 5‐point ranking scale.

O \ S Rank	1	2	3	4	5 (Score)
**1**	I	I	II	II	III
**2**	I	II	II	III	III
**3**	I	II	II	III	III
**4**	I	II	III	III	III
**5**	III	III	III	III	III

**TABLE A2:** Risk matrix of failure modes by occurrence (O) and severity (S) ranking, with classwise risk distribution for TG‐100 10 point scale.

O \ S Rank	1 (1–2)	2 (2–3.5)	3 (3.5–5)	4 (6–8)	5 (9–10) (Score)
**1 (1‐2)**	I	I	II	II	III
**2 (2‐3.5)**	I	II	II	III	III
**3 (3.5‐5)**	I	II	II	III	III
**4 (6‐8)**	I	II	III	III	III
**5 (9‐10)**	III	III	III	III	III

**TABLE A3:** Risk matrix of failure modes by occurrence (O) and severity (S) ranking, with classwise risk distribution for Non‐TG‐100 10 point scale.

O \ S Rank	1 (1)	2 (2, 3)	3 (4, 5, 6)	4 (7, 8)	5 (9, 10) (Score)
**1(1)**	I	I	II	II	III
**2(2, 3)**	I	II	II	III	III
**3(4, 5, 6)**	I	II	II	III	III
**4(7, 8)**	I	II	III	III	III
**5(9, 10)**	III	III	III	III	III

## APPENDIX B

### Supplementary results

Supplementary data to this article can be found at the following link:

FMEASystematicEvaluation.xlsx FMEASystematicEvaluation.xlsx
